# Active Substances from the Micro-Immunotherapy Medicine 2LC1^®^ Show In Vitro Anti-Cancer Properties in Colon, Prostate, and Breast Cancer Models and Immune-Enhancing Capabilities in Human Macrophages

**DOI:** 10.3390/ijms26094300

**Published:** 2025-05-01

**Authors:** Camille Jacques, Irene Marchesi, Francesco Paolo Fiorentino, Flora Marchand, Mathias Chatelais, Ilaria Floris

**Affiliations:** 1 Preclinical Research Department, Labo’life France, Pescalis-Les Magnys, 79320 Moncoutant-sur-Sevre, France; ilaria.floris@labolife.com; 2 Kitos Biotech s.r.l.s., Porto Conte Ricerche, S.P. 55 Porto Conte-Capo Caccia, Km 8,400 Loc. Tramariglio, 07041 Alghero, Italy; iremarchesi@gmail.com (I.M.); fpfiorentino@gmail.com (F.P.F.); 3 ProfileHIT, 7 Rue du Buisson, 44680 Sainte-Pazanne, France; flora.marchand@profile-hit.com (F.M.); mathias.chatelais@profile-hit.com (M.C.)

**Keywords:** micro-immunotherapy, low doses, ultra-low doses, macrophages, cancer cells models, C-MYC, macrophages

## Abstract

Tumor-associated macrophages (TAMs) play a pivotal role in cancer regulation by influencing tumor growth, metastasis, and the immune microenvironment. By providing low doses and ultra-low doses (ULD) of immune regulators to the organism, micro-immunotherapy (MI) medicines (MIM) could be seen as valuable adjuvant drugs in the context of a wide range of pathological conditions, including cancers. Thus, these MIM could target TAMs, affecting their phenotype and activities. In this study, the anti-tumor and the immune-stimulatory effects of four capsules out of the ten composing the Labo’life’s MIM 2LC1^®^ (2LC1-1, 2LC1-6, 2LC1-7, and 2LC1-8), as well as the specific nucleic acid (SNA^®^) sequence SNA-MYC present at ULD in this medicine have been evaluated in vitro, in several cancer models, and in human monocyte-derived macrophages. Our results showed that the tested MI formulations increased the tumor cell death of spheroids from HCT-116 colon cancer cells, while reducing the spheroid volume. Moreover, the treatments impaired the clonogenic capabilities of two cancer cell lines from epithelial origin, the LNCaP prostate cancer and the MCF-7 breast cancer cells. Interestingly, ULD of the SNA-MYC shared similar anti-cancer capabilities in those models, and it led to a significant reduction in the expression of C-MYC when evaluated in a model of human M2 macrophages. In the same model, the MI formulations also increased the expression of CD86 and HLA-DR, two markers of M1 anti-tumor macrophages. In addition, the tested items modulated the secretion of a panel of chemokines related to macrophage activity and immune cell recruitment. Finally, our results showed that 2LC1-8 increased the phagocytosis capabilities of human monocyte-derived macrophages, thus possibly contributing to sustaining the immune functions of M1, which are crucial in the context of cancer. Even if more research is needed to uncover their exact mechanism of action, these results suggest that the tested capsules of 2LC1 as well as ULD of SNA-MYC display both anti-tumor and immune-enhancing effects.

## 1. Introduction

Cancer remains one of the leading causes of death worldwide. Despite significant advances in conventional treatment, there is an urgent need to explore complementary therapeutic approaches that can improve treatment effectiveness and reduce undesirable side effects, thus enhancing the quality of life for patients. As cancer can be considered an immune-mediated disease [1], a better understanding of the role of the immune system (IS) in the regulation of tumor initiation, formation, growth, and progression is pivotal for therapy. This knowledge will shape the future development of cancer immunotherapy and facilitate its integration with conventional treatments. In line with these considerations, the role played by cytokines has been widely investigated in the context of cancer. Cytokines facilitate cell–cell communication, playing essential roles in immunity and in promoting or inhibiting cell differentiation and proliferation [2]. While cytokines are crucial for coordinating inflammatory responses within the tumor microenvironment (TME), immunotherapies employing cytokines such as interferon (IFN)s and interleukin (IL)s, are recognized as potential cancer therapies [2]. Historically, cytokines have been utilized as anti-cancer drugs for more than fifty years, with IFN-α and IL-2 being the first to be employed [3,4]. 

The micro-immunotherapy (MI) medicinal products of Labo’life, also called MI medicines (MIM), employ low doses (LD) and ultra-low doses (ULD) of immune mediators, mostly cytokines, as well as nucleic acids and chemicals, to support the IS in different contexts, including cancers [5]. The active ingredients, obtained according to a homeopathic procedure, are expressed in terms of decimal Hahnemannian dilutions (DH), centesimal Hahnemannian dilutions (CH), or Korsakovian dilutions (K). Depending on the dilution, the active ingredients are sensed to orient a particular biological response towards either an activation or an inhibition [6,7]. A previously published study reported the anti-tumor properties of a MIM employing LD and ULD of cytokines such as IFN-α and IL-2 in association with other actives, like tumor necrosis factor (TNF)-α at 5 CH, specifically in the context of colorectal cancer (CRC) [5]. To pursue the investigations on MIM in the framework of CRC and other cancer types, we focused on the 2LC1^®^ medicine (further referred as 2LC1). This medicine also employs LD or ULD of IFN-α and IL-2 in association with other actives, such as TNF-α at 5 CH, and it could be seen as an interesting immunotherapy adjuvant drug in the context of cancer treatments. 2LC1 is composed of ten different capsules, each one with its specific combination of active ingredients impregnated into pillules (also called globules) for an oromucosal administration. This medicine, as with all of the MIM from Labo’life, is intended to be sequentially administered, (capsule-1 first, then capsule-2, etc.), one capsule at a time, each day or several times per day depending on the needs. The presence of ULD of specific nucleic acids (SNA^®^) also referred to here and in our previous articles as SNA, is another specificity of MIM.

In this first pilot study, only four capsules out of the ten composing 2LC1 were tested. Based on their position in the sequence, they are 2LC1-1, 2LC1-6, 2LC1-7, and 2LC1-8. In particular, 2LC1-1 and 2LC1-8 employ TNF-α (5 CH). All of them employ ULD of SNA-MYC to target the product of the proto-oncogene *cellular myelocytomatosis* (*c-myc*), an oncoprotein involved in the development and progression of a wide range of cancers, including CRC, prostate cancer (PC), and breast cancer (BC) [8,9,10,11,12,13,14,15]. To investigate the effects of ULD of SNA-MYC, two unitary products were produced for research purposes: SNA-MYC (18 CH) and SNA-MYC (10 CH). In particular, 2LC1-6 is the only capsule employing the SNA-MYC (18 CH), while the others use this SNA at 10 CH. 

On the other hand, and in addition to ULD of SNA, the ULD of growth factors, such as transforming growth factor-β (TGF-β) are also employed in 2LC1. Growth factors are widely known players involved in cancer progression [16], and especially TGF-β, which displays a tumor-promoting role during the late and advanced stages of cancer, due to its effects on epithelial to mesenchymal transition, cell migration/invasion, and immune suppression [17]. Transposed to the clinical setting, stage I-III BC patients with high expression of TGF-β receptor II (TβRII), TβRI and TβRII, and p-Smad2, and low expression of Smad4 had an unfavorable prognosis concerning progression-free survival, delineating here the importance of TGF-β pathway in BC [18]. In CRC, elevated levels of TGF-β within the TME are recognized as a key hallmark of immune evasion, leading to T-cell exclusion and hindering the development of the Th1 effector phenotype [19]. 

2LC1 capsules also employ ULD of epidermal growth factor (EGF). In particular, 2LC1-7 uses EGF at 15 CH. Interestingly, TGF-β has been shown to work synergistically with EGF in promoting cancer invasion and metastasis [20,21]. Indeed, while recent research indicates that EGF receptor (EGFR) could be a crucial target in treating triple-negative BC, basal-like BC, and inflammatory BC [22], the interplay between EGF and TGF-β signaling has been reported as equally important in PC [23,24]. Finally, as C-MYC up-regulation is amplified by EGFR signaling in murine models of CRC [25], the combined use of ULD of TGF-β, EGF, and SNA-MYC in 2LC1 is intended to downregulate their associated pathways, counteracting the uncontrolled cancer cell proliferation. 

From an immune perspective, M1 macrophages play a pivotal role in cancer prevention due to their pro-inflammatory and anti-tumor activities. These macrophages are characterized by markers such as CD86 [26], and human leukocytes antigen (HLA)-DR [27], and are involved in the destruction of tumor cells and the inhibition of tumor growth. M1-type macrophages secrete chemokines such as CXCL3 for instance [28], which not only sustains their M1 macrophages differentiation [29], but also contributes to the recruitment of other immune cells to the tumor site, thus creating an environment hostile to cancer progression. In contrast, M2 macrophages, also known as tumor-associated macrophages (TAMs), are often detrimental to cancer patients. M2 macrophages support tumor growth and metastasis by promoting tissue remodeling, angiogenesis, and immune suppression, and overexpress C-MYC in comparison to M1 macrophages [30,31]. Their presence in the TME is associated with poor prognosis, as they facilitate tumor evasion. Therefore, shifting the balance from M2 to M1 could be seen as an interesting strategy in developing effective cancer therapies. In particular, evidence reported that the M2c macrophages supports tumor growth and their circulating number is correlated with disease severity [32], as the percentage of M2c-like macrophages in BC patients was significantly higher in advanced stages [33].

In this study, the potential role of 2LC1-1, 2LC1-6, 2LC1-7, 2LC1-8, and two active ingredients present in these formulations, SNA-MYC (10 CH) and SNA-MYC (18 CH), in targeting carcinogenesis and immune mechanisms was appraised in vitro. Thus, the tested MI products were investigated in their ability to influence: (i) tumor cell proliferation and viability in a three-dimensional (3D) model of CRC cells, (ii) the clonogenic capacity of PC and BC cell lines, (iii) C-MYC expression and the M2c/M1-like switch in phenotype in human peripheral blood mononuclear cells (PBMCs)-derived macrophages. In addition, 2LC1-8 was tested for its phagocytosis capabilities in human monocyte-derived macrophages, an important anti-tumor mechanism of defense of the IS.

## 2. Results

### 2.1. Actives from 2LC1 Display Cytotoxic and Anti-Proliferative Effects in a Three-Dimensional In Vitro Model of Colon Cancer Spheroids

The spheroid cancer model, when compared to the conventional two-dimensional culture system, better reflects the in vivo situation of tumors. While only cancer cells can form spheroids, this is not a feature shared by all tumor cell lines. In particular, spheroids formed with the CRC cell line HCT-116 are considered a suitable model to screen compounds and drugs with potential anti-cancer effects [34]. Thus, this three-dimensional in vitro model was used to assess the cytotoxic effects of 2LC1 on spheroids under starvation conditions (1% fetal bovine serum [FBS]). In this first experimental model, only three capsules of 2LC1 were tested, according to an experimental protocol that has previously been described [5]. 

**Figure 1:**
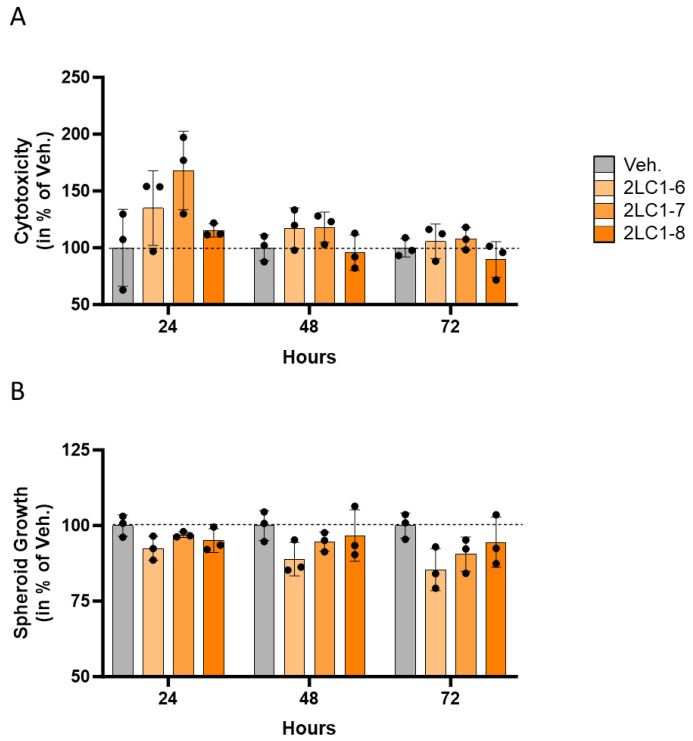
The tested complex MI formulations 2LC1-6, 2LC1-7, and 2LC1-8 display cytotoxic and anti-proliferative effects in a 3-dimensional in vitro model of CRC spheroids. (**A**) Spheroids were cultivated in starvation conditions (1% FBS) and treated for 24, 48, or 72 h with either the Veh., 2LC1-6, 2LC1-7, or 2LC1-8. The cytotoxicity of the treatments was evaluated thanks to the CellTox green fluorescent dye. The cytotoxicity percentages results reflect the mean normalized green fluorescence intensity (NGFI) ± standard deviation (S.D.) from CellTox green fluorescent dye, for each condition (*n* = 3). NGFI was calculated as described in Section 4.2.3. (**B**) Effects of 2LC1-6, 2LC1-7, and 2LC1-8 were assessed on HCT-116-derived spheroid growth in 1% FBS. The spheroid volume was calculated for each treatment condition at either 24-, 48- or 72 h, and expressed as a fold change in each endpoint measure, normalized to the initial spheroid volume at Day 0. Results are presented as the mean percentages ± S.D. of the Veh.-treated spheroids for each time point. Each condition was performed in *n* = 3 technical replicates (black dots). The dotted black lines are drawn to highlight the effect of the tested formulations compared with the Veh.

The results presented in Figure 1A illustrate the cytotoxicity of the tested capsules towards the HCT-116-spheroids, as a percentage of the Veh., for each time point. At 24 h the strongest effect was found, with increased cytotoxicity of about 35%, and 70%, in 2LC1-6 and 2LC1-7-treated spheroids, respectively, when compared to the Veh. On the other hand, 2LC1-8 did not show any effect at this time point. At 48 h, 2LC1-6 and 2LC1-7 were the only capsules that had an effect in increasing the cytotoxicity (of about 15–20%) in comparison with the Veh. Finally, after 72 h of incubation, the overall cytotoxic effect disappeared, suggesting that there is a time-dependent effect that progressively decreases after 48 and 72 h. Overall, these results highlighted a time-dependent reduction in the cytotoxic effect of the 2LC1-6, 2LC1-7, and 2LC1-8 capsules, thus revealing that, in the assessed conditions, those items were most likely able to affect the viability of cancer cells forming spheroids after 24 h of incubation. 

In parallel to the cytotoxicity, the anti-proliferative effects of the 2LC1 capsules were appraised in the same experiment. Thus, the spheroids’ growth was monitored, and the results are presented in Figure 1B. After 24 h of incubation, a slight reduction in the spheroid volume (of about 5–10%) was noticed, in all tested conditions. After 48 h of incubation, the effect stayed about the same as after 24 h. Finally, after 72 h of incubation, the overall effects of the tested capsules were found higher than the precedent tested time points. Among all the tested capsules, 2LC1-6 seemed the one that displayed the more pronounced effect in reducing spheroid growth. Overall, these results showed that, in 1% FBS conditions, the tested items reduced the spheroid growth in comparison with the Veh. The effect was seen in all time points, and it seems to increase in a time-dependent manner. 

The impact of the 2LC1 capsules on the cytotoxicity and the spheroid growth was also evaluated in 10% FBS, and the same respective upwards and downwards trends were reproduced, even if the magnitude of the effect was smaller (Supplementary Figure S1A,B). The complete sequential medicine has been tested in vivo on the growth of HCT-116 cell-derived xenografted tumors, as previously described [5]. At the end of the treatment, a trend toward a decrease in the tumor volume was observed in the 2LC1-treated group compared with the Veh. one (*p* = 0.1; Supplementary Figure S2A). Indeed, with median primary tumor volumes of 1800 mm^3^ in the Veh. group and 1267.5 mm^3^ in the 2LC1 one, the treatment induced a median decrease of about 30%. These results are in line with the tumor weight measures, made at the time of euthanasia (Supplementary Figure S2B), as a trend toward a reduction was also found (*p* = 0.79). The median weights of the tumors were indeed 1.42 g in the Veh. group vs. 1.06 g in the 2LC1 group, which corresponds to a decrease of about 25%. Both trends suggest a possible effect in reducing the tumor growth of HCT-116 CRC cells, when subcutaneously engrafted in nude mice. 

Altogether, the overall data obtained in HCT-116 cells indicated that the capsules 2LC1-6, 2LC1-7, and 2LC1-8 could exert cytotoxic and anti-proliferative effects that merit further investigations.

### 2.2. Actives from 2LC1 Reduced the Clonogenic Capabilities of Cancer Cells

**Figure 2:**
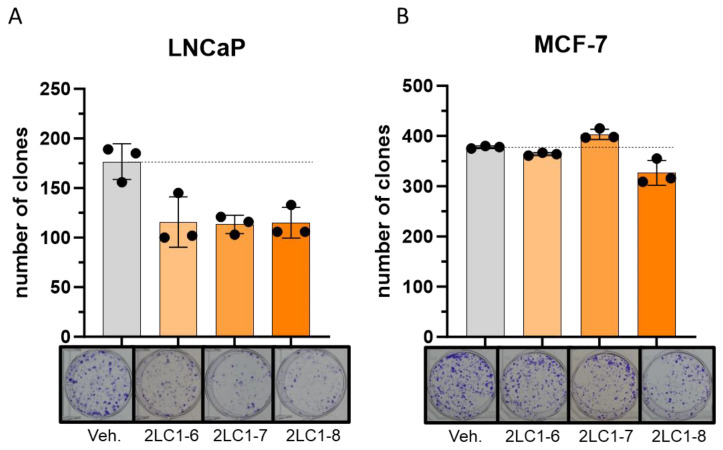
The tested complex MI formulations 2LC1-6, 2LC1-7, and 2LC1-8 reduce the clonogenic capabilities of cancer cells. Two cancer cell lines from epithelial origin, (**A**) LNCaP (PC) and (**B**) MCF-7 (BC), were cultivated for 72 h in the presence of either the Veh., 2LC1-6, 2LC1-7, or 2LC1-8 before being tested for their clonogenic capabilities. (Upper panel): the data are presented as the mean ± S.D. of the number of clones counted at the end of the 10 days incubation period, after Crystal Violet staining. The experiment has been performed in *n* = 3, with each dot representing the cell count obtained in one well of the triplicate. (Lower panel): representative pictures of the wells obtained after clones’ staining, taken thanks to a binocular magnifier. The dotted black lines are drawn to highlight the effect of the tested MI formulations when compared with the Veh.

In an attempt to assess if 2LC1-6, 2LC1-7, and 2LC1-8, could impact the tumorigenic features of cancer cell types other than CRC, a clonogenic experiment was performed in two cancer cell lines: the PC cells LNCaP and the BC cells MCF-7. Our results showed that, in the LNCaP cells, all three tested formulations, when incubated for 72 h, displayed similar magnitudes in terms of their effects, as they all reduced the cells’ clonogenic capabilities by about 35%, when compared with the Veh. (Figure 2A). However, the effects of these items were more heterogeneous in the MCF-7 BC cells. Indeed, while 2LC1-6 did not show an effect in comparison with the Veh., 2LC1-7 slightly increased the clones’ number by about 6%, and 2LC1-8 reduced this number by about 15% (Figure 2B). In addition, and as presented in Supplementary Figure S3, when assessed in the MNNG-HOS osteosarcoma cell line (non-epithelial cells), none of the tested capsules displayed any inhibitory effect on the clonogenic capabilities, which were rather increased in these cells, under the tested experimental conditions. Altogether, these data highlighted that the three capsules were able to similarly reduce the clonogenic capacity of LNCaP, while only 2LC1-8 was able to consistently impede the clonogenic capabilities of two tumor cell lines from epithelial origin, LNCaP and MCF-7.

### 2.3. SNA-MYC (18 CH), Displayed Anti-Proliferative and Anti-Clonogenic Properties in Cancer Cells

**Figure 3:**
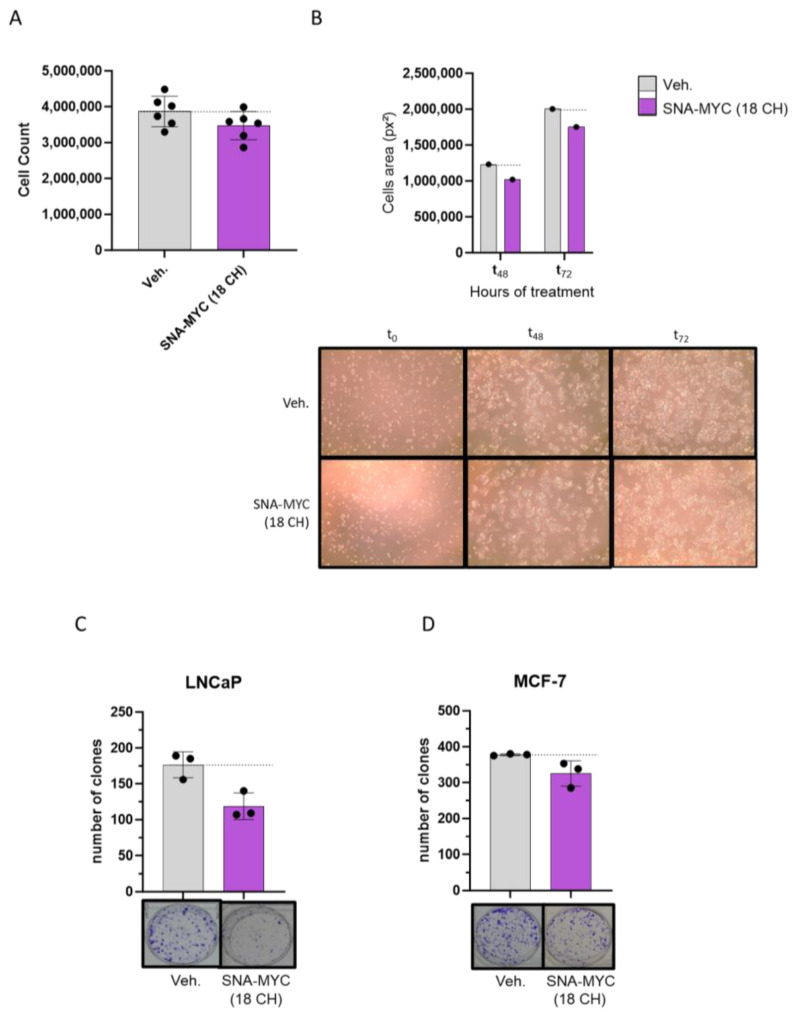
One of the actives from the tested MI formulations 2LC1, the unitary SNA-MYC (18 CH), displays anti-proliferative and anti-clonogenic properties in cancer cells. Colon cancer cells from HCT-116 cell line were starved for 24 h, without FBS supplementation, and were then subjected to either a Veh., or a SNA-MYC (18 CH) treatment, in 1% FBS, during 72 h. (**A**) At the end of the incubation period, live cells were counted by the Trypan Blue method. The results present the total amount of live cells counted in each condition (*n* = 6), and are illustrated as the mean ± S.D. (**B**) Pictures of the wells were taken during the course of the experiment: at the initial plating time (t_0_); after 48 h (t_48_); and after 72 h (t_72_), thanks to an optical magnifier (×4 magnification). The total area covered by the cells in each picture was quantified by ImageJ software, and the results are presented, in each treatment condition, at t_48_ and t_72_ (upper panel). The quantization has been performed once, and the results are expressed in px^2^, for the Veh., and the SNA-MYC (18 CH) treatments (gray vs. purple histograms, respectively), at each time point. The analyzed pictures are presented (×4 magnification) in the (lower panel). (**C**,**D**) The tested SNA-MYC (18 CH) reduces the clonogenic capabilities of cancer cells. Two cancer cell lines from epithelial origin, (**C**), LNCaP (PC), and (**D**) MCF-7 (BC), were cultivated during 72 h in the presence of either the Veh., or SNA-MYC (18 CH), before being tested for their clonogenic capabilities. Upper panel: the data are presented as the mean ± S.D. of the number of clones counted at the end of the 10-day incubation period, after Crystal Violet staining. The experiment has been performed in *n* = 3, each dot representing the cell count obtained in one well of the triplicate. Lower panel: representative pictures of the wells obtained after clones’ staining, taken thanks to a binocular magnifier. The dotted black lines are drawn to highlight the effect of the tested SNA-MYC (18 CH) when compared with the Veh.

SNA-MYC (18 CH), one of the active ingredients included in 2LC1, was selected to address its effects, when employed alone, in similar cancer models. Thus, the CRC cell line HCT-116 was used, and the cells were starved for 24 h (without FBS supplementation), before undergoing a 72-h treatment with either the Veh., or SNA-MYC (18 CH), in 1% FBS. At the end of the incubation period, live cells were counted by the Trypan Blue method, and the results are presented in Figure 3A. In such conditions, a reduction in the cell count of about 10% was observed in the SNA-MYC (18 CH) treatment condition, when compared to the Veh. In addition, in this experiment, pictures of the wells were also taken at the time of cell plating (t_0_), after 48 h (t_48_), and after 72 h (t_72_) of incubation, and the cell proliferation was evaluated by image analysis, through the determination of the area occupied by the cells in each condition. Figure 3B (upper panel) shows, for each condition, the area occupied by the cells, on the analyzed images, after calculation through ImageJ software. The corresponding pictures are illustrated in Figure 3B (lower panel). After 48-h as well as after 72 h of treatment with SNA-MYC (18 CH), a similar reduction in the area covered by the HCT-116 cells was observed, of about 15%, in comparison with the Veh. Thus, these results could be interpreted as an effect of the SNA-MYC (18 CH) in reducing the proliferative capabilities of the HCT-116 cells. The same clonogenic experiment as previously performed (see previous Section 2.2) was conducted with the SNA-MYC (18 CH). Interestingly enough, the clonogenic capabilities of the LNCaP were reduced by about 30% (Figure 3C), and the ones of the MCF-7 were reduced by about 15% (Figure 3D) by the SNA-MYC (18 CH) in comparison with the Veh. On the other hand, about 15% more clones were formed, as a consequence of SNA-MYC (18 CH)-treatment, in comparison with the Veh, in the MNNG-HOS cell line (Supplementary Figure S4). Taken together, this set of experiments suggested that the SNA-MYC (18 CH) displays anti-proliferative properties in vitro, in HCT-116 cells, under starvation, and anti-clonogenic features in PC and BC cells. In addition, these results show coherence with the above-presented results of 2LC1 capsules as both unitary and complex MI formulations employing ULD of SNA-MYC displayed anti-cancer properties in HCT-116, LNCaP, and MCF-7 models.

### 2.4. Actives from 2LC1 Reduced the Expression of C-MYC in a Model of Human CD14^+^-Derived M2c Macrophages

The next experiment aimed to investigate the effect of SNA-MYC used either at 10 or 18 CH on the expression of C-MYC. C-MYC is reported to be involved in the tumorigenic properties of cancer cells [35], and it is also able to influence the phenotype and the pro-tumoral features of TAMs [36]. In particular, as M2c macrophages are involved in tumor progression [32], human CD14^+^-derived M2 macrophages were employed to assess how the tested formulations could influence these cells’ phenotype. M2 macrophages display higher C-MYC expression than M1 [30], and this was confirmed in our experiment as the IL-10-polarized M2c macrophages used as control expressed higher C-MYC levels, in comparison with IFN-γ-polarized-M1 ones (Supplementary Figure S5). Then, when M2c macrophages were switched towards a M1 profile (“M2c > M1” control condition), they expressed a level of C-MYC that was intermediate between the M2c and the M1 subtypes. In this experiment, 2LC1-1, 2LC1-6, and 2LC1-8, as well as the two unitary MI formulations, SNA-MYC (10 CH) and SNA-MYC (18 CH) were tested.

**Figure 4:**
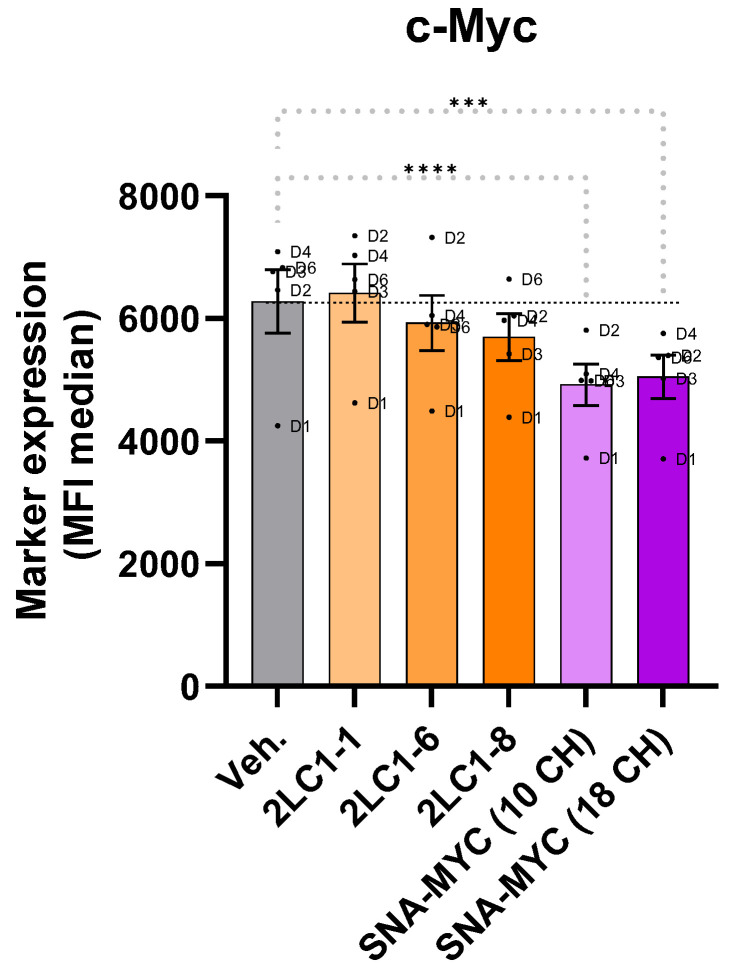
Three complex MI formulations from 2LC1, as well as SNA-MYC employed at 10 CH and at 18 CH, reduce the expression of C-MYC, in a model of human CD14^+^-derived M2c macrophages. Briefly, PBMCs were retrieved from *n* = 6 healthy blood donors (D1–D6), separated on Ficoll and cultivated in the presence of macrophage colony-stimulating factor (M-CSF; 50 ng/mL) and 20 ng/mL IL-10, in order to make them differentiate into M2c macrophage subtype. Media were renewed on day 5 and the capsules of 2LC1, along with SNA-MYC (10 CH), SNA-MYC (18 CH), and the Veh., were incubated with the macrophages for the next three days. A boost of lipopolysaccharide (LPS; 100 ng/mL) was administered on day 7 and the cells were detached, permeabilized, and immune-stained for the analysis of C-MYC expression by flow cytometry, on day 8. The results are presented as the mean ± standard error of the mean (S.E.M.) of median fluorescence intensity (MFI) values obtained for *n* = 5 donors (D5 has been excluded from the analysis, due to too few collected cells). Each dot represents the values obtained as the mean of a duplicate measure for each donor. The dotted black lines are drawn to highlight the effect of the tested items, when compared with the Veh. One way ANOVA, **** *p* < 0.0001, *** *p* < 0.001, compared with the Veh.-treated M2c macrophages.

Interestingly, SNA-MYC (10 CH) and SNA-MYC (18 CH) both significantly inhibited C-MYC expression compared to the Veh. (Figure 4). In addition, 2LC1-6 and 2LC1-8 displayed an inhibitory effect on the expression of C-MYC compared to the Veh. Of note, none of the tested capsules impacted the viability of the M2c macrophages (Supplementary Figure S6). Altogether, these data could mean that the two actives of 2LC1, SNA-MYC (10 CH) and SNA-MYC (18 CH), are able to reduce the expression of the oncoprotein C-MYC, in the high-C-MYC-expressing human M2c-macrophages.

### 2.5. Actives from 2LC1 Increased the Expression of CD86 and HLA-DR

**Figure 5:**
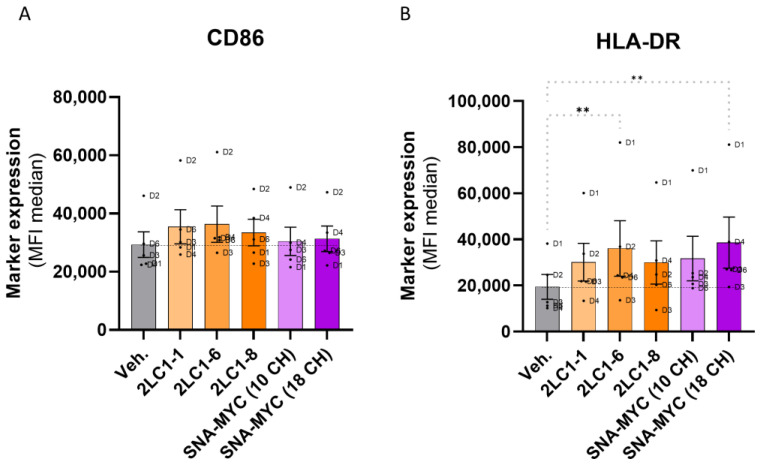
Three complex MI formulations from 2LC1, as well as the unitary MI formulations, SNA-MYC employed at 10 CH and at 18 CH increase the expression of CD86 and HLA-DR, in a model of human CD14^+^-derived M2c macrophages. Briefly, PBMCs were retrieved from *n* = 6 healthy blood donors (D1–D6), separated on Ficoll and cultivated in the presence of M-CSF (50 ng/mL) and 20 ng/mL IL-10, in order to make them differentiate into M2c macrophage subtype. Media were renewed on day 5 and the tested capsules of 2LC1-1, 2LC1-6, 2LC1-8, SNA-MYC (10 CH), SNA-MYC (18 CH), and the Veh., were incubated with the macrophages for the next three days. A boost of LPS (100 ng/mL) was administered on day 7 and the cells were detached and immune-stained for the analysis of (**A**), CD86, and (**B**), HLA-DR expression, by flow cytometry, on day 8. The results are presented as the mean ± S.E.M. of MFI values obtained for *n* = 5 donors (D5 has been excluded from the analysis, due to too little collected cells). Each dot represents the values obtained as the mean of a duplicate measure for each donor. The dotted black lines are drawn to highlight the effect of the tested items, when compared with the Veh. One-way ANOVA, ** *p* < 0.01 compared with the Veh.-treated M2c macrophages.

In order to complement the first set of data, the effect of the MI formulations from 2LC1 on macrophages activation markers was appraised in the same model of human CD14^+^-derived M2c macrophages. As CD86 and HLA-DR are two markers that are expressed by anti-tumor M1 macrophages [37], their expression was at first appraised in M2c, in “M2c > M1”-switched, and in M1 control macrophages, to confirm that the expression of these markers was higher in M1 than in M2c (Supplementary Figure S7). In addition, when treated with 2LC1-1, 2LC1-6, 2LC1-8, SNA-MYC (10 CH), or SNA-MYC (18 CH), a slight increase in the expression of CD86 was found (Figure 5A). The same upward trend was found for HLA-DR expression, the results even reaching statistical significance for SNA-MYC (18 CH) (Figure 5B). Altogether, these results, even if they still need to be confirmed, could mean that actives from 2LC1 may induce changes in the expression of CD86 and HLA-DR, which could reflect a possible effect in promoting a shift from the pro-tumoral, M2-type TAM phenotype to the anti-tumoral M1-type one.

### 2.6. Actives from 2LC1 Increased the Secretion of Chemokines in a Model of Human CD14^+^-Derived M2c Macrophages

**Figure 6:**
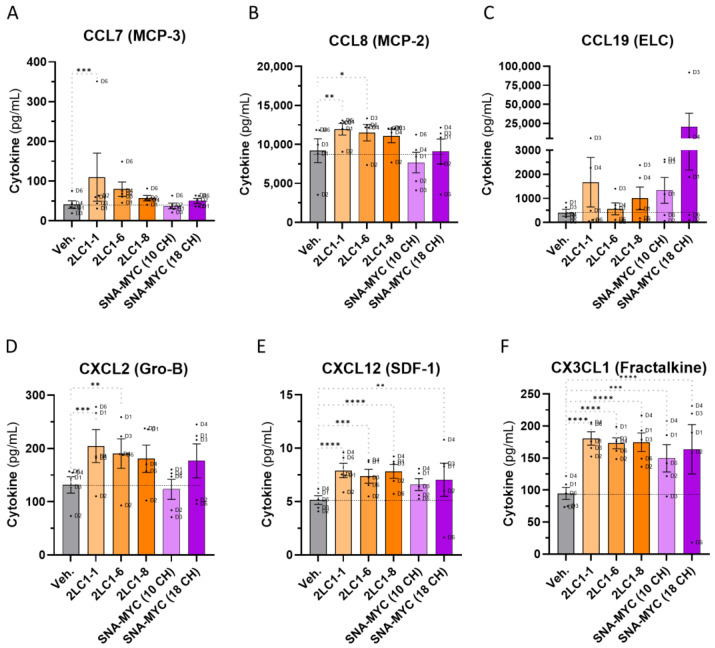
Three complex MI formulations from 2LC1, as well as the unitary MI formulations, SNA-MYC employed at 10 CH and at 18 CH, increase the secretion of several chemokines in a model of human CD14^+^-derived M2c macrophages. Briefly, PBMCs were retrieved from *n* = 6 healthy blood donors (D1–D6), separated on Ficoll and cultivated in the presence of M-CSF (50 ng/mL) and 20 ng/mL IL-10, in order to make them differentiate into M2c macrophage subtype. Media were renewed on day 5 and the tested capsules of 2LC1-1, 2LC1-6, 2LC1-8, SNA-MYC (10 CH), SNA-MYC (18 CH), and the Veh., were incubated with the macrophages for the next three days. A boost of LPS (100 ng/mL) was administered on day 7 and the SNs were collected on day 8 for multiplex assay. (**A**–**F**) The secretion levels of (CCL7 (MCP-3), CCL8 (MCP-2), CCL19 (ELC), CXCL2 (Gro-B), CXCL12 (SDF-1), and CX3CL1 (Fractalkine)) were thus quantified. The results are presented as the mean pg/mL ± S.E.M. of the values obtained for *n* = 5 donors (D5 has been excluded from the analysis due to too little collected cells). Each dot represents the values obtained as the mean of a duplicate measure for each donor. The dotted black lines are drawn to highlight the effect of the tested items when compared with the Veh. One way ANOVA, **** *p* < 0.0001, *** *p* < 0.001, ** *p* < 0.01, * *p* < 0.05 compared with the Veh.-treated M2c macrophages.

Cytokines and chemokines are crucial in shaping the TME, affecting immune cell recruitment and cancer progression. As macrophages are key contributors to this microenvironment, and given the prior findings that the tested capsules of 2LC1 may promote an M1 profile, assessing their effect on the cytokines and chemokines secreted by those cells is essential. Thus, in the same experimental setting as previously described, the secretion of seven cytokines (TNF-α, IL-4, IL-6, IL-12p40, IL-23, IL-1Ra, and IP-10), and six chemokines (CCL7 (MCP-3), CCL8 (MCP-2), CCL19 (ELC), CXCL2 (Gro-B), CXCL12 (SDF-1), and CX3CL1 (Fractalkine)) has been assessed in the supernatants (SNs) after treatment with either 2LC1-1, 2LC1-6, 2LC1-8, SNA-MYC (10 CH), or SNA-MYC (18 CH) (Figure 6A–F). The tested items did not affect or just barely reduced the secretion of the tested cytokines, without reaching significance, except for TNF-α (Supplementary Figure S8). Of note, the tested items increased the secretion of the tested chemokines in comparison with the Veh., with the only exception of SNA-MYC (10 CH), which did not affect the levels of CCL7, CCL8, and CXCL2 (Figure 6A,B,D). Interestingly, the secretion of CX3CL1 was importantly increased by all the tested items in a significant manner (Figure 6F), and the one of CXCL12 was augmented by four out of the five tested items (Figure 6E). Altogether, these results indicate that the tested complex MI formulations, SNA-MYC (10 CH) and SNA-MYC (18 CH), increased the secretion of chemoattractant factors in a model of treated human CD14^+^-derived M2c macrophages. These results, even if they still need to be confirmed and further tested, open perspectives on the possibility that the active substances employed in 2LC1 could modulate macrophage responses by inducing an up-regulation in the secretion of some chemokines.

### 2.7. 2LC1-8 Enhances the Phagocytic Capabilities of Macrophages In Vitro

**Figure 7:**
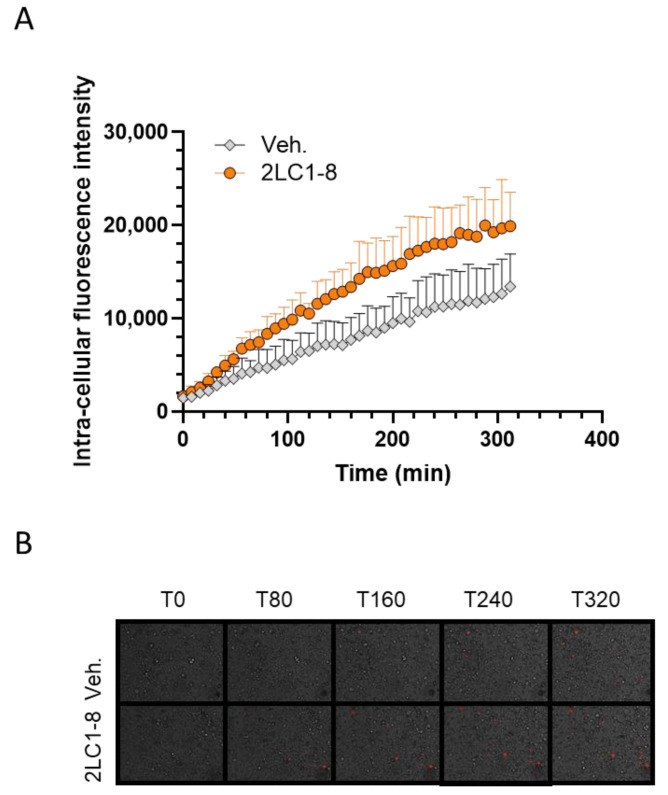
2LC1-8 increases the phagocytosis capabilities of human monocyte-derived macrophages in vitro. (**A**) Monocytes isolated from one healthy donor were differentiated into macrophages for 7 days in the presence of GM-CSF and IFN-γ. The treatment with either the vehicle (Veh.) or 2LC1-8 started 24 h before inducing the phagocytosis. Each condition was performed in *n* = 6 replicates. The data are presented as the mean ± S.D. (**B**) Representative pictures of the wells at different time points (80 min (T80), 160 min (T160), 240 min (T240) and 320 min (T320)). Macrophages are visualized in bright light, phagocytosed *C. albicans* corresponding to the red spots.

Regarding the paramount importance of the immune reactions in cancer progression and treatment, and especially those mediated by macrophages [38], we finally wanted to assess the effects of 2LC1-8 on the activation of macrophages. This capsule employs TNF-α (5 CH) together with IFN-α (6 CH), along with other actives at 10 CH, characteristics that are shared with another MI formulation, which was shown to enhance the phagocytosis of macrophages [39]. In light of these previously published results and because 2LC1-8 was the capsule that mostly impacted the expression of C-MYC, CD86, and HLA-DR in M2c macrophages (Figure 4 and Figure 5A,B), and the secretion of CXCL12 and CX3CL1 (Figure 6E,F), two chemokines that are implicated in the phagocytic features of the macrophages [40,41], the last part of the study, solely focused on this capsule. In the experimental protocol, previously described [39], human monocytes isolated from one healthy donor were cultivated for seven days in a macrophage-differentiating medium containing granulocyte-macrophage colony-stimulating factor (GM-CSF) and IFN-γ, and treated for 24 h with either the Veh., or 2LC1-8. The cells were then pre-incubated with heat-killed-pHRodo™ Red-labeled-*C. albicans* for 30 min and fluorescence was then monitored every 8 min over 6 h. As the pHRodo™ Red-dye emits fluorescence in an acidic milieu only, an increase in the intensity of the signal over time would attest to a proper dye penetration within the phagosome, thus reflecting the phagocytosis activity. The results are presented in Figure 7A,B. The rate of phagocytosis slightly increased over time in the Veh.-treated condition (gray dots), and in the 2LC1-8-treated condition (orange dots), and it could also be observed that 2LC1-8 induced a higher level of phagocytosis than the Veh. Indeed, after about 300 min, the phagocytosis capabilities were almost 50% more elevated in the 2LC1-8-treated macrophages than in the Veh.-treated ones. In addition, the phagocytosis-enhancing capacity of 2LC1-8 was also appraised in another experimental model, in which human granulocytes were preincubated with the tested items for 10 min before the adjunction of fluorescent beads to the culture medium, for the next 45 min. The phagocytosis of the beads was monitored by flow cytometry, at the end of the 55 min incubation period. In those conditions, no difference was observed between the Veh. and the 2LC1-8 capsule (Supplementary Figure S9A,B). Apart from the difference in the cell models used, a pre-incubation of only 10 min applied in granulocytes, instead of 24 h, may have played a role, explaining the main reason no effect was observed. Overall, these results suggested that 2LC1-8 displayed a time-dependent stimulatory effect on the phagocytic capability of human macrophages in vitro.

## 3. Discussion

Considering that malignant cells (i) succeed in evading detection and destruction by immune defenses, and that (ii) long-term infections and inflammation, characterized by restricted or polarized immune reactions, play a role in the development of cancer and the advancement of tumors, cancer can be considered as an immune-mediated disease [1].

The IS is the first line of defense against malignant cells, and it plays an important role at each phase of cancer progression, from initiation to metastatic spreading. The idea of targeting and boosting immune responses in the context of cancer treatment has decades of experience in clinics and cancer immunotherapy is a still-growing and expanding therapeutic approach holding promising perspectives [42]. The MIM from Labo’life could offer beneficial effects as adjuvant drugs in this context. In particular, a sequential MI medicine employing active substances also used in 2LC1, displayed anti-cancer properties in the context of CRC [5]. 

As this study wanted to pursue the investigations on the possible mode of action and potential of these medicines, specific combinations of active substances at LD and ULD were selected. Thus, the current study aimed to evaluate the effects of complex MI formulations corresponding to specific capsules composing the sequential MIM 2LC1. Two unitary MI formulations produced for research purposes have also been included in the analysis: SNA-MYC (18 CH) and SNA-MYC (10 CH). These are ULD-based active ingredients also present in 2LC1 composition.

The research approach could be seen with a double axis as it considered both aspects of the TME; (i) the cancer cells themselves, and (ii) the immune cells, especially the macrophage sub-population. Concerning the evaluation of the tested items in cancer models, our in vitro data highlighted that 2LC1-6, 2LC1-7, and 2LC1-8 displayed anti-tumor effects in a 3D model of HCT-116 CRC cells, as they reduced the volume of spheroids while displaying a cytotoxic effect (Figure 1A,B). 

In addition, the tested capsules also reduced the clonogenic capabilities of several other cancer cell lines from epithelial origin, including the LNCaP PC and the MCF-7 BC cells (Figure 2A,B). Finally, our results also revealed that the SNA-MYC, when used alone as a unitary preparation, reduced the viability of HCT-116 (Figure 3A,B), and decreased the clonogenic capabilities of LNCaP and MCF-7 cells (Figure 3C,D). 

The TME plays a crucial role in regulating tumor growth and metastatic spreading. Among the types of immune cells recruited to the tumor area, macrophages are the most abundant and can be found at all stages of tumor progression. In solid tumors, these cells could even comprise up to 50% of the tumor mass [43]. From a biological standpoint, macrophages undergo a process called M1/M2 polarization, influenced by signals such as cytokines present within the TME, leading to different phenotypes, divergent cell specialization, and functional features. While M1 macrophages can inhibit tumor growth through their immune-boosting effects, their M2 counterparts are more likely to have pro-tumoral effects by favoring tumor growth, angiogenesis, metastasis, and resistance to existing treatments. An additional layer of complexity has been added to this simplistic phenotypic dichotomy, as various subsets of the M2 have been identified, known as M2a, M2b, M2c, and M2d, which are induced by different stimuli and exhibit distinct phenotypes and functions. Moreover, evidence supports that all these M2 subtypes have pro-tumoral effects [32]. Thus, one of the goals of this study was to assess the effect of certain actives from the MIM 2LC1 on macrophages, focusing first on the M2c phenotype. In light of our results, the tested formulations influenced these cells by reducing the M2c pro-tumoral profile, while sustaining the immune-boosting effects of M1 macrophages. Although further investigations are needed, this body of data is promising regarding the potential of this therapy in the context of certain cancers. 

In addition, our study also aimed at evaluating the effects of some of the actives from 2LC1 on the expression level of C-MYC. This transcription factor regulates various cellular processes such as cell proliferation, apoptosis, cell survival, tissue remodeling, angiogen-esis, cell metabolism, and the production of inflammatory and anti-inflammatory cyto-kines. Additionally, it plays a role in cell transformation [44]. It is worth noting that, in human macrophages studied in vitro, the expression of C-MYC is limited to the M2 phenotype and is almost undetectable in resting M0 and in pro-inflammatory M1 macrophages [30]. Thus, our flow cytometry analysis confirmed that the expression levels of C-MYC in M2c macrophages were higher than in M1 macrophages. Our results reported that SNA-MYC (10 CH) and SNA-MYC (18 CH), as well as 2LC1-6, and 2LC1-8, reduced the expression of C-MYC compared to the Veh. (Figure 4). These results support the down-regulatory effect of ULD-SNA-MYC on their intended matching target, C-MYC. While very preliminary, these results support the rationale behind the use of ULD of SNA-MYC, providing additional explanations of the current results. For example, a reduction in the clonogenic capabilities of PC cells has already been reported as a consequence of a daunomycin-conjugated triplex-forming oligonucleotides c-myc-targeting [45]. Thus, the downregulation of C-MYC observed here in macrophages could be driven by the two ULD-based SNA-MYC unitary products. While being cautious with these assumptions, if this effect was transposed in our cancer cell models, it could have, in turn, affected the clonogenic capabilities of the PC and BC cells (Figure 3A,B). In the future, it could thus be valuable to assess the effect of those two ULD-based SNA-MYC on the expression of C-MYC in cancer cell models to validate this hypothesis. In addition, the inclusion of a ULD-based scramble with similar chemical characteristics to SNA will be considered to provide proper experimental controls in forthcoming experiments.

From both sides of (i) cancer cells and (ii) macrophages within the TME, those results are quite interesting. Indeed, (i) strategies aiming at targeting C-MYC in cancer cells have shown promising effects, especially on clonogenic capabilities. Moreover, (ii) put into the context of cancer and the so-called TAMs, the effect of the tested MI capsules may be linked to an “anti-TAM” effect. Indeed, the density of TAMs is linked to poor prognosis, and strong evidence also indicates that TAMs accelerate cancer progression and metastasis [46]. These M2 polarized cells indeed possess powerful immunosuppressive functions, exhibiting poor antigen-presenting capabilities and inhibiting T-cell activation and dendritic cell functions. 

Thus, in an attempt to connect the immune side effects of the tested MI formulations to a tumor-related microenvironment, and regarding the fact that all the M2-types macrophages, especially the M2c subtype, were shown to display pro-tumoral effects [32], we wanted to address if the MI formulations could revert the pro-tumoral M2c profile to an anti-tumor M1 profile. Based on the characterization of the M2c as bona fide TAMs, due to (i) their high capacity to produce pro-tumoral factors and (ii), their involvement in tumor escape through angiogenesis and extra-cellular matrix degradation facilitation [32], a particular emphasis was placed on this subset as our model of choice. Therefore, the capability of 2LC1-1; -6; -8, the SNA-MYC (10 CH) and the SNA-MYC (18 CH) to possibly revert such M2c subtype to the more anti-tumor M1 one was appraised. Overall, our results showed that the tested MI formulations could enhance the expression of CD86 and HLA-DR (Figure 5A,B), and especially 2LC1-6 and SNA-MYC (18 CH), which significantly increased the levels of HLA-DR (Figure 5B). Increased expression of co-stimulatory molecules has largely been reported in macrophages in the context of infections, and particularly, the overexpression of CD86 was found able to enhance the ability of THP-1 macrophages to defend against *Talaromyces marneffei* [47]. In the context of cancer, our results are quite interesting, as a strategy that could overcome the tumor-induced immuno-suppressive environment is crucial for effective immunotherapy. As M2c macrophages are known for their immunosuppressive and tissue-remodeling roles, and as they typically exhibit low expression of co-stimulatory molecules like CD86 and major histocompatibility complex (MHC)-II proteins such as HLA-DR, the reprogramming of a more pro-inflammatory and antigen-presenting phenotype through MIM could be promising. Indeed, such phenotypic M2-M1 shift may enhance the ability of macrophages to present tumor antigens to T-cells and stimulate a stronger adaptive immune response against cancer cells. By enhancing the antigen presentation capability and co-stimulatory signals, the tested MI actives could improve the efficacy of anti-tumor immune responses, potentially leading to better control or the eradication of tumors. To dig deeper into the effects of the tested MI formulations on the functions of the TAMs-related M2c polarized macrophages, an assessment of the secreted chemokines has been performed in the same model. Understanding the secreted chemokines profile helps in several ways: in our case, it can directly reveal if the tested actives of 2LC1 can influence macrophage polarization, and from a practical standpoint, could be extended to immune-related side effects predictions and guidance in the choice of combinatory therapeutic approaches. Nonetheless, our results seemed to display an overall effect of the tested items in increasing the secretion levels of CCL7 (MCP-3), CCL8 (MCP-2), CCL19 (ELC), CXCL2 (Gro-B), CXCL12 (SDF-1), and CX3CL1 (fractalkine) (Figure 6A–F). All those factors have specific roles and immune cells’ recruitment functions and, regarding CCL7, our results reported that 2LC1-1 was the most powerful out of all the tested items in inducing an increase in the secretion of this chemokine (Figure 6A). This may be explained, at least partially, by its composition, as 2LC1-1 is the only one containing IL-1β (6 CH) and TNF-α (5 CH). Further studies on these two MI actives are ultimately needed; however, it is interesting to report here that, when associated, IL-1β and TNF-α elicited an immediate response, resulting in the increased expression of CCL7 by fibroblasts, epithelial cells, and endothelial cells [48]. CCL-8 is known for its implication in the migration of monocytes and T-cells, leading to an anti-tumor response. Furthermore, in a model of cutaneous squamous cell carcinoma Ji et al. found that 5-aminolevulinic acid-mediated photodynamic therapy upregulated CCL8 expression, elevated the number of macrophages within the tumor, and promoted their M1 pro-inflammatory phenotype [49]. Such findings could corroborate our results about the possible effect of the tested items in modulating the expression of some markers and cytokines characterizing the M2c in a more M1-type phenotype (Figure 5A,B and Figure 6B). Interestingly enough, an in-depth screening of the chemokines that differentially induce chemotaxis of M1 and M2 macrophages revealed that CCL19 only induced M1 chemotaxis, and induced the activation of both mitogen-activated protein kinase kinase 1 (MEK1)-extracellular signal-regulated kinase 1/2 (ERK1/2) and phosphatidylinositol 3 kinase (PI3K)-protein kinase B cascades in M1, but not in M2 macrophages [50]. In light of our results (Figure 6C), this study is very interesting, as, even if non-significant and heterogeneous in their magnitude depending on the MI item tested, all of them displayed an ability to enhance the secretion of CCL19, which could lead to a strengthened recruitment of M1 macrophages instead of M2. Concerning CXCL2, our results highlighted that 2LC1 actives, and especially 2LC1-1 and 2LC1-6, increased the secretion of this factor (Figure 6D). Interestingly, the study conducted by Rouault et al. in visceral white adipose tissue from obese subjects reported that CXCL2 mRNA positively correlated with M1-type markers but not with M2-type macrophage markers, and that the secretion of CXCL2 was stimulated in a more pronounced extent by M1-LPS-treated-monocyte-derived macrophages than M2-dexamethasone-treated cells [51]. CXCL12 is well-known to attract immune cells such as NK cells, T-cells, and neutrophils, all of which express its receptor, CXCR4 [52]. Using the CXCR4 antagonist, AMD3100, and a neutralizing anti-CXCL12 antibody in a model of urinary tract infection, Isaacson et al. confirmed that the disruption of the CXCL12/CXCR4 interaction significantly decreased the accumulation of these immune cells at the site of infection. The importance of immune cells communication through chemokines is now well established, and this feature has been of paramount interest for the outcome of PC [53] and BC treatment [54]. Thus, the increase in the secretion of CXCL12 that was induced by all the tested MI items in vitro (Figure 6E) may be another valuable effect. Finally, regarding the CX3CL1 results (Figure 6F), the significant increase in its secretion that was observed in all the MI treatment conditions could be seen as a response of macrophage stimulation. Of this concern, it is interesting to cite the study from Gong et al., indicating that CX3CL1 overexpression accelerated LPS-induced macrophage activation [55]. In addition, such an increase in macrophages activation and in CX3CL1 expression could be beneficial for the patients, as a high expression of this factor is an indicator of good prognosis for CRC [56]. Accordingly, it has been reported that an increased expression of CX3CL1 sustained anti-tumor responses by favoring the infiltration of NK cells, T-cells, and dendritic cells into the tumor [57,58]. In line with these observations, it can be concluded that therapeutic strategies aimed at increasing the expression of this chemokine are promising [59,60,61]. Notably, the increased CX3CL1 secretion induced by all the tested actives of 2LC1, if further confirmed, may offer clinical benefits in the context of cancer. 

Moreover, from an immune perspective, our results showed that the formulation of 2LC1-8 enhanced the phagocytosis capabilities of human monocyte-derived macrophages in a time-dependent manner (Figure 7A,B), thus possibly contributing to the maintenance of the immune functions, which are crucial in the context of cancer [1]. The specific com-position of the capsule 2LC1-8, which employs TNF-α (5 CH), IFN-γ (6 CH), and other actives could, at least partially, explain our observations (see Table 1). Indeed, a previous study of ours already reported the effect of one MI formulation containing human recombinant IFN-γ (6 CH) and TNF-α (5 CH), among other actives, in increasing the phagocytic capacities of macrophages, in a time-dependent manner, under the same experimental conditions [39]. 

Globally this study provides encouraging evidence regarding the potential anti-tumor and immune-stimulatory properties of several 2LC1 capsules, and two of their actives; SNA-MYC (10 CH) and SNA-MYC (18 CH), on cancer cells and macrophages. Nevertheless, there are several important limitations to consider. One can be represented by the small number of cancer types included in this study, which restricts the generalizability of the findings. Each cancer type has its own genetic and epigenetic characteristics, making it necessary to perform specific in vitro assays to test the medicines. Broadening the diversity of tumor models could thus improve the understanding of MIM efficacy across various cancer types. Expanding the study to include a broader array of tumor types/genetic backgrounds would also allow the identification of specific contexts in which the MIM is most effective. In addition, the study primarily focused on measuring specific markers, such as CD86, HLA-DR, and C-MYC, which, while informative, do not comprehend the full spectrum of immune interactions and pathways that might be involved. A more comprehensive investigation into additional molecular pathways might provide insights into the mechanisms of action of the MIM 2LC1, including the SNA targets. Finally, the lack of statistical power for some of the experiments performed here should be addressed in the future.

Finally, to overcome the limitations associated with these in vitro investigations, future research should delve deeper into in vivo studies in suitable models under which the tested MIM might exert its multi-target and synergistic effect on the IS, TME, and cancer cells. Investigations should explore the systemic and local effects, with a particular focus on TME related factors. Research into potential combination therapies with existing cancer treatments may also reveal strategies to maximize therapeutic benefits. This comprehensive approach will be crucial in advancing the application of MIM such as 2LC1 as an adjuvant therapy in oncology.

## 4. Materials and Methods

### 4.1. Tested Items and Experimental Controls

The tested items are as follows: (i) two unitary products SNA-MYC (18 CH) and SNA-MYC (10 CH) that were produced for research purposes, and (ii) four out of the ten capsules of 2LC1 (2LC1-1, 2LC1-6, 2LC1-7, and 2LC1-8). Their composition is listed in Table 1. All the tested items and the vehicle (Veh.) capsules were manufactured and provided by Labo’life España, as previously described [6,39]. For the in vivo part of the study, all ten capsules of the MIM were sequentially administered to the animals. 

The Veh. control sugar globules used in the study and in previously published studies are produced to provide a suitable control for preclinical research [6,39,62,63,64]. These sugar globules are the same control for all the tested capsules of 2LC1. For the current in vitro tests, the sugar globules of 2LC1 or the Veh. were freshly diluted either in 50 mL or in 100 mL of fresh culture medium, to reach the final sucrose-lactose concentration of 22 mM or 11 mM.

### 4.2. Colon Cancer In Vitro Model

#### 4.2.1. Initiation of the Spheroid Model

HCT-116 cells were obtained from the American Type Culture Collection (ATCC, Manassas, VA, USA). HCT-116 CRC cells were cultured in Dulbecco′s Modified Eagle′s medium (DMEM) (ref: D5671, Sigma-Aldrich, Saint-Louis, MI, USA) supplemented with 10% FBS (Euroclone, Pero, Italy; #ECS0180L), 2 mM L-glutamine (ref: X0550, VWR International, Radnor, PA, USA), antibiotic antimycotic solution (ref: L0010, VWR International), sodium pyruvate (ref: S8636, Sigma-Aldrich) and non-essential amino acids (ref: M7145, Sigma-Aldrich). Cells were incubated at 37 °C, 5% CO_2_ humidified air. For the 3D spheroid experiment, cells were detached by trypsinization and suspended in fresh phenol red-free complete culture medium (Euroclone, LOBE12917F), either in standard conditions (10% FBS), or in starvation conditions (1% FBS), depending on the experiments. Then, 20 μL of cell suspension, containing 500 cells, were plated in each well of a 384-well black, clear round bottom ultra-low attachment spheroid microplate (Corning 3830, Corning, NY, USA). Plating of cells was performed with an automated liquid handling platform (Gilson Pipetmax, Gilson Incorporated, Middleton, WI, USA). Plates were incubated at 37 °C and 5% CO_2_ humidified air to allow spheroid formation. After 72 h of incubation, spheroids were used for cytotoxicity or growth assay and were incubated with either the Veh., 2LC1-6, 2LC1-7, or 2LC1-8, for the next 72 h. During the treatment course, the final FBS percentages were either kept at 1% or 10%.

#### 4.2.2. Kinetics of 3D Spheroid Cytotoxicity and Growth

Ten μL of fresh medium containing either the Veh., 2LC1-6, 2LC1-7, or 2LC1-8, at proper dilution, supplemented with CellTox green fluorescent dye (Promega, Madison, WI, USA) were used to treat the spheroids. As a non-toxic DNA stain, CellTox green dye is excluded from viable cells but stains the dead cells’ DNA. When the dye attaches to DNA in damaged cells, its fluorescent properties increase, making the fluorescent signal generated by the dye correspond to the quantity of dead cells. Three technical replicates were performed. An automated liquid handling platform was used for the preparation and treatment of spheroids (Gilson Pipetmax, Gilson Incorporated). Immediately after the start of treatment, plates were incubated at 37 °C and 5% CO_2_ in an automated digital widefield microscope with temperature and CO_2_ control system (BioTek Cytation 5, BioTek Instruments, Winooski, VT, USA). Using a 4x objective, three brightfield and GFP fluorescence images at different z-axis levels were captured immediately at the beginning of the treatment and then at 24-, 48-, and 72 h post-treatment.

#### 4.2.3. Data Analysis

Brightfield and green fluorescent protein (GFP) images at distinct z-axis (50 μm between each image) were processed and merged to obtain 3D images covering 150 μm of the z-axis. Z-projected brightfield images were used to define the spheroid area (further used for the determination of the spheroid volumes). The Green Fluorescence Integral (GFI) within the defined spheroid area, emitted by the CellTox green dye (Promega) and indicative of cell death, was quantified using Z-projected GFP images. These values were later used for cytotoxicity representations, shown as normalized green fluorescence intensity (NGFI).

Spheroid volume at each time point (V) was calculated as follows:

$$V=A\times \frac{4}{3}\sqrt{\frac{A}{\pi }}$$

where A is the measured area of the spheroid at the same time point.

Spheroid growth at each time point was calculated as: V/initial V.

NGFI of each spheroid, at each time point, was calculated as:

$$\mathrm{NGFI}=\frac{GFI-{GFI}_{0}}{A}$$

where GFI is green fluorescence integral at the analyzed time point, GFI_0_ is green fluorescence integral of the same spheroid at time point zero (immediately after start of treatment), and A is the area of the same spheroid at the analyzed time point [65,66].

The results for spheroid growth and NGFI were expressed as a percentage relative to the Veh.-treated cells, which were set at 100%.

### 4.3. Colon Cancer Animal Model

All the information related to the in vivo protocol are described in the previously published study [5].

### 4.4. Evaluation of the Clonogenic Capabilities

MNNG-HOS (osteosarcoma), LNCaP (PC), and MCF-7 (BC) cell lines were procured from the ATCC, and their clonogenic capabilities were evaluated. Briefly, 250,000 cells/well were plated in simplicate in 6-well-plates, in DMEM added with 2.5% FBS (for the MCF-7 and the MNNG-HOS), or in Roswell Park Memorial Institute (RPMI) medium added with 10% FBS (for the LNCaP cells), on day 0 (D0). On day 1 (D1), the cells were treated with either the Veh., 2LC1-6, 2LC1-7, 2LC1-8, or SNA-MYC (18 CH), at the final sucrose–lactose concentration of 11 mM. On day 2 (D2), and day 3 (D3), the treatments were renewed with the same capsules, diluted in a fresh medium. On day 4 (D4), treated cells from each condition were harvested, and re-plated at 1000 cells/well, in 6-well-plates, in triplicates, in the appropriate medium added with 10% FBS, without any MI treatment. The cells were finally incubated, without any MI treatment until the end of the experiment, with the medium being renewed every two days, and stained with Crystal Violet on day 14 (D14).

### 4.5. Evaluation of the Anti-Proliferative Activity of the SNA-MYC (18 CH) in Colon Cancer Cells

HCT-116 cells were cultured in DMEM high-glucose supplemented with 2 mM L-glutamine, AA solution (100 units/mL penicillin, 10 μg/mL streptomycin, 25 ng/mL amphotericin B), with 1% FBS. The assay measured the number of viable cells after 24 h of serum starvation and 72 h of treatment in medium with 1% serum. On day 0 (D0), the cells were suspended in complete medium and counted using Cell Countess II (Life Technologies, Carlsbad, CA, USA). A total of 100,000 cells were plated in 3 T25-flasks with vented filters. The next day, the medium was removed and substituted with 5 mL of complete medium without FBS (starvation). After 24 h of serum starvation, the medium was substituted with 5 mL of medium 1% FBS supplemented with either the Veh., or SNA-MYC (18 CH), at the final sucrose–lactose concentration of 11 mM. The cells were kept treated for 72 h; the treatments/media being renewed every day. At the end of the incubation, the cells were detached by trypsinization and counted with Trypan Blue assay using Cell Countess II (Life Technologies). During the course of the treatment (t_0_, t_48_, and t_72_), pictures were taken with a microscope (magnification ×4), and the area covered by the cells was evaluated by ImageJ software (version 1.53k), and expressed in px^2^, in each treatment condition, at t_48_ and t_72_.

### 4.6. Evaluation of the Macrophages Profiles

Peripheral blood mononuclear cells (PBMCs) were retrieved from *n* = 6 healthy blood donors (D1–D6) at the Etablissement Français du Sang (EFS). The PBMCs were isolated from 2 tubes of blood (EDTA tubes, EFS Nantes) by centrifugation on a Ficoll gradient (density 1.077 g/mL). The PBMCs ring was collected, washed in phosphate-buffered saline (PBS), and then the pellet was resuspended in RPMI 1640 for automatic counting with the LUNA-II^TM^ system (Logos Biosystems, Villeneuve d’Ascq, France), including cell viability measurement. Cells were plated at a density between 282,000 and 641,000 cells/well, depending on the donors, and incubated for 3.5 h (day 0). At the end of the incubation period, adherent cells were carefully washed and cultured in RPMI 1640 medium supplemented with 2% human serum (HS, Sigma-Aldrich), non-essential amino acids (1 mM, Lonza Bâle, Switzerland), pyruvate (1 mM, Lonza), L-glutamine (2 mM, Sigma-Aldrich), 4-(2-hydroxyethyl)-1-piperazineethanesulfonic (HEPES) buffer (10 mM, Lonza), and M-CSF (50 ng/mL, PeproTech Inc., Thermo Fisher Scientific, Waltham, MA, USA). For the different macrophages profiles (M1 control or M2c control), the following inducers were added the next day (day 1): 20 ng/mL IFN-γ (M1), 20 ng/mL IL-10 (M2c), in order to make them differentiate into the macrophage subtype of choice. Media were renewed on day 5 with the same cocktail of cytokines for the controls. In the case of the switch controls “M2c > M1”, 20 ng/mL IFN-γ was added on day 5 instead of the following tested capsules 2LC1-1, 2LC1-6, 2LC1-8, SNA-MYC (10 CH), SNA-MYC (18 CH), and the Veh., which were tested at a final concentration of 11 mM. The content of one capsule was diluted in 12.5 mL of RPMI 1640 and after solubilization for 45 min in a water bath, the solution was diluted appropriately and applied to the cells without prior filtration. The compounds were incubated with the PBMCs-derived macrophages for three days at 37 °C, starting from day 5. A boost of LPS (100 ng/mL) was administered on day 7 (for 24 h) to facilitate cytokine detection in the SNs. On day 8, the SNs were collected and frozen for subsequent measurement of 13 cytokines/chemokines (TNF-α, IL-4, IL-6, IL-12p40, IL-23, IL-1Ra, IP-10, CCL-7 (MCP-3), CCL-8 (MCP-2), CCL-19 (ELC), CXCL2 (Gro-B), CXCL12 (SDF-1), CX3CL1 (fractalkine)). The cells were then detached and dissociated for multiparametric immunostaining (CD86/HLA-DR/C-MYC/Zombie), with signal acquisition performed by flow cytometry (NLS, CYTEK, 3-laser VBR configuration). As the final number of collected cells was too small, the data from the donor 5 (D5) were not included in the analysis.

### 4.7. Phagocytosis Capabilities Assessment

#### 4.7.1. In Human Monocytes-Derived Macrophages

Peripheral blood mononuclear cells from healthy volunteers were obtained from EFS after being isolated from buffy coats and standard Ficoll-Hypaque gradient method. Monocytes were isolated from PBMCs by adherence to plastic for 2 h in macrophage serum-free medium (M-SFM, Gibco) optimized for macrophage culture, at 37 °C in a humidified atmosphere containing 5% CO_2_. Monocytes were seeded and differentiated in macrophages in the presence of GM-CSF and IFN-γ, provided by Peprotech. *C. albicans* was heat-killed (1h at 75 °C) before labeling with pHRodo™ Red, succinimidyl ester (pHRodo™ Red, SE, ThermoFisher Scientific), according to the manufacturer’s instructions. The pHRodo dye is a non-fluorogenic molecule at neutral pH, drastically becoming fluorescent in an acidic milieu. This situation happens in phagolysosome, thus indicating an effective phagocytosis. *C. albicans* was introduced in sodium bicarbonate with pHRodo™ dye (final concentration of 20 μg/mL) for 1 h, RT, in the dark. The excess of dye was washed with PBS. Then, pHRodo™ Red-labeled *C. albicans* was finally diluted in PBS and left at 4 °C until use. The Control for correct labeling was realized in an acid solution (pH = 4) vs. neutral solution (pH = 7.4). After 7 days of monocyte differentiation into macrophages, Veh. or 2LC1-8 were incubated for 24 h with the cells at the final sucrose–lactose concentration of 22 mM. The next day, the culture medium and its treatments were replaced, before pHRodo™-labeled *C. albicans* was added to the cells and left on ice to allow the proper *C. albicans*’ sedimentation. After 30 min on ice, the plate was read on an Operetta apparatus for 6 h at 37 °C, 5% CO_2_, and data acquisition was realized thanks to Harmony^®^ Imaging Software (Perkin Elmer, Waltham, MA, USA). Each well was imaged every 8 min and the Columbus 2.5.0 image analysis software was used to do the phagocytosis quantitation. The experiment was carried out once and six replicates were run for all conditions.

#### 4.7.2. In Human Granulocytes

Granulocytes were isolated from the total peripheral blood of a healthy donor following Ficoll gradient separation. The cells were cultured in RPMI-1640 medium supplemented with 2 mM L-glutamine, 100 U/mL penicillin, 100 μg/mL streptomycin, and 0.1% bovine serum albumin (BSA) at 37 °C with 5% CO_2_. The experiment was conducted in 96-well plates where cells from one donor were treated either with the Veh. or with 2LC1-8 (at concentrations of 11 mM or 22 mM), pre-incubated with the cells for 10 min at 37 °C. Fluorescent beads (Molecular Probes™ FluoSpheres™ Carboxylate-Modified Microspheres, 1.0 μm, yellow-green fluorescent 505/515) were added and incubated with the cells for an additional 45 min. Untreated cells not incubated with beads served as a negative control. All conditions were performed in triplicate. After incubation, the cells were rinsed with PBS/BSA 0.1% and centrifuged. Data acquisition was performed using 10,000 cells per replicate on a BD FACSVerse™ cytometer. Considering the increase in fluorescence in the fluorescein isothiocyanate (FITC) channel corresponds to the number of phagocytosed beads (with an emission wavelength of 515 nm), results were initially expressed as percentages of FITC-positive cells, and then as a percentage relative to the Veh.-treated cells, which were set at 100%.

### 4.8. Statistical Analysis

The graphs in the figures were performed with GraphPad Prism, Version 10.2.3.403 for Windows (GraphPad Software Inc., San Diego, CA, USA, accessed on 30 April 2024). The authors have adhered to recent recommendations that advocate for the use of descriptive statistics in fundamental research rather than statistical inferences when dealing with a limited number of independent values [67]. According to these guidelines, if results are obtained from only one, two, or three (*n* = 1, or *n* = 2, or *n* = 3) experiment(s), it is preferable to present the complete dataset, plot the data points, and allow readers to interpret the findings themselves rather than drawing statistical inferences, showing *p* values, S.D., or S.E.M. As such, no statistical inference has been conducted on the majority of the study results presented here. The one-way ANOVA has solely been performed on those data consisting of *n* = 6 independent values derived from six donors (see Section 4.4, Section 4.5 and Section 4.6).

## 5. Conclusions

In this study, the anti-tumor and immune-stimulatory effects of four MI complex formulations from the MIM 2LC1 (the capsules 2LC1-1, 2LC1-6, 2LC1-7, and 2LC1-8) have been evaluated in vitro across various cancer models, including 3D spheroid models of HCT-116 CRC cells, classical 2D cultures of LNCaP PC cells, and MCF-7 BC cells, as well as in human monocyte-derived macrophages. In these cancer models, the tested 2LC1 capsules exhibited a cytotoxic effect on CRC cell spheroids and decreased their volume. Additionally, the treatments influenced the clonogenic capabilities of two epithelial cancer cell lines: LNCaP PC and MCF-7 BC. Furthermore, our results related to the SNA-MYC, one of the ingredients used at ULD in the tested capsules, demonstrated that this active, when used alone, exhibited similar anti-cancer capabilities in vitro, effectively reducing the proliferation and the number of clones of those cancer cells. Interestingly, when appraised in a model of CD14^+^-derived M2 macrophages model, the 2LC1 capsules reduced C-MYC expression and increased the one of CD86 and HLA-DR, likely indicating a shift from the pro-tumoral M2c-TAMs-related phenotype to a more M1-associated phenotype, with enhanced anti-tumor properties. Moreover, in the same model, the tested MI formulations resulted in a global increase in the secretion of chemokines such as CCL7, CCL8, CCL19, CXCL2, CXCL12, and CX3CL1, suggesting the potential of these active substances to support the recruitment of other immune cells within the TME. Lastly, from an immune-related standpoint, our results showed that 2LC1-8 increased the phagocytosis capabilities of human monocyte-derived macrophages in a time-dependent manner, potentially contributing to the maintenance of immune functions that are critical in the context of cancer. Altogether, these first set of results indicate that the tested actives and capsules of 2LC1 could display both anti-tumor and immune-enhancing effects.

## Figures and Tables

**Table 1:** Composition of the four tested capsules 2LC1-1, 2LC1-6, 2LC1-7, and 2LC1-8. The actives used in each capsule are mentioned as “Starting Materials” and are expressed in CH.

Starting Materials (CH)	2LC1-1	2LC1-6	2LC1-7	2LC1-8
hr-IL-1β	6	10	10	10
hr-IL-2	10	10	10	10
hr-IL-4	10	10	10	10
hr-IL-6	6	10	10	10
hr-IL-7	10	10	10	10
hr-IFN-α	10	10	10	10
hr-IFN-γ	10	10	10	6
Dimethylsulfoxyde	10	10	3	10
hr-EGF	10	10	15	10
hr-TGF-β	10	10	30	10
hr-GM-CSF	10	10	10	10
hr-TNF-α	5	10	10	5
SNA-HLA-I	10	10	10	10
SNA-HLA-II	10	10	10	10
DNA (K)	10	10	12	30
RNA (K)	6	10	10	10
SNA-C1a	10	10	10	10
SNA-C1b (including SNA-MYC)	10	18	10	10

CH: centesimal Hahnemannian; DNA: deoxyribonucleic acid; EGF: epidermal growth factor; GM-CSF: granulocyte-macrophage colony-stimulating factor; HLA: human leukocyte antigen; hr: human recombinant; IFN: interferon; IL: interleukin; K: Korsakovian dilution; RNA: ribonucleic acid; SNA: specific nucleic acid; TGF-β: tumor growth factor-β; TNF-α: tumor necrosis factor-α.

## Data Availability

The data of the current study are available from the corresponding author upon reasonable request.

## Supplementary Materials

The following supporting information can be downloaded at: https://www.mdpi.com/article/10.3390/ijms26094300/s1.

## Abbreviations

The following abbreviations are used in this manuscript:

3D: three-dimensional; BC: breast cancer; BSA: bovine serum albumin; c-myc: cellular myelocytomatosis; CH: centesimal Hahnemannian; CRC: colorectal cancer; DH: decimal Hahnemannian dilutions; DNA: deoxyribonucleic acid; DMEM: Dulbecco’s Modified Eagle’s medium; EFS: Etablissement Français du Sang; EGF: epidermal growth factor; EGFR: epidermal growth factor receptor; ERK 1/2: extracellular signal-regulated kinase 1/2; FBS: fetal bovine serum; FITC: fluorescein isothiocyanate; GFI: green fluorescence integral; GFP: green fluorescent protein; GM-CSF: granulocyte-macrophage colony-stimulating factor; HLA: human leukocyte antigen; hr: human recombinant; IFN: interferon; IL: interleukin; IS: immune system; K: Korsakovian dilution; LD: low doses; LPS: lipopolysaccharide; M-CSF: macrophage colony-stimulating factor; MEK1: mitogen-activated protein kinase kinase 1; MHC: major histocompatibility complex; MI: micro-immunotherapy; MIM: micro-immunotherapy medicine; M-SFM: macrophage serum-free medium; NGFI: normalized green fluorescence intensity; NK: natural killer; PBMCs: peripheral blood mononuclear cells; PBS: phosphate-buffer saline; PC: prostate cancer; PI3K: phosphatidylinositol 3-kinase; RNA: ribonucleic acid; RPMI: Roswell Park Memorial Institute; S.D.: standard deviation; S.E.M.: standard error of the mean; SNA: specific nucleic acid; SNs: supernatants; TAMs: tumor associated macrophages; TβR: TGF-β receptor; TGF-β: transforming growth factor-β; TME: tumor microenvironment; TNF-α: tumor necrosis factor-α; ULD: ultra-low doses; Veh.: vehicle.
