# Supplementary material for: Active Substances from the Micro-Immunotherapy Medicine 2LC1® Show In Vitro Anti-Cancer Properties in Colon, Prostate, and Breast Cancer Models and Immune-Enhancing Capabilities in Human Macrophages
Source: Int J Mol Sci. 2025 May 1;26(9):4300. doi: 10.3390/ijms26094300 (PMC12072473; doi:10.3390/ijms26094300)
Supplement: Supplementary file 1 [file ijms-26-04300-s001.zip › ijms-3594152-supplementary.pdf]

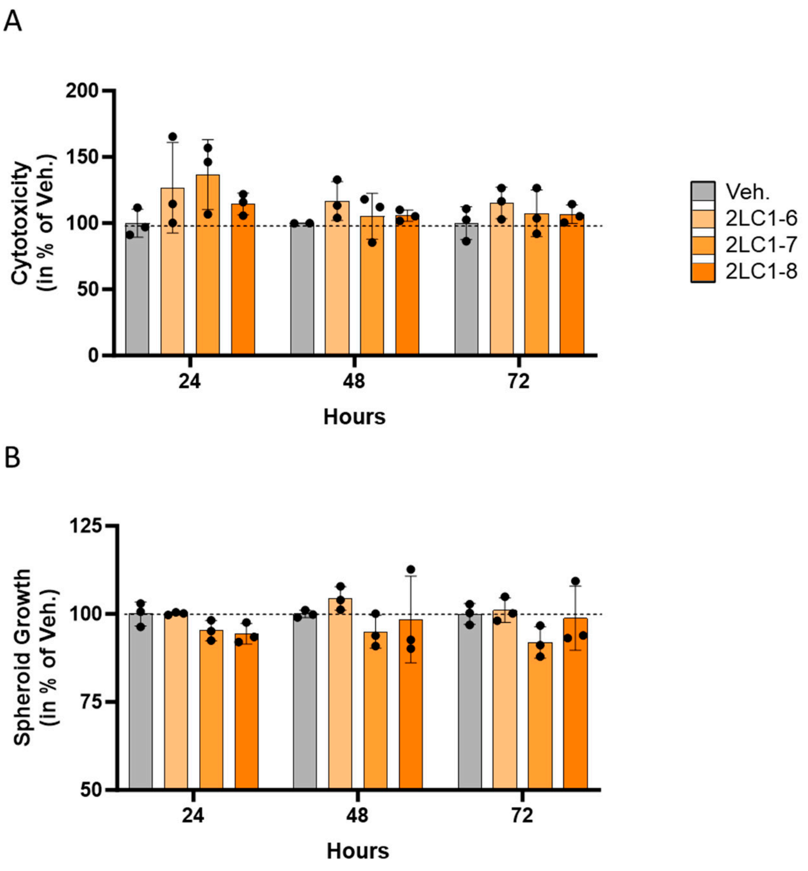

Supplementary Figure S1: The tested complex MI formulations 2LC1-6, 2LC1-7 and 2LC1-8 display cytotoxic and anti-proliferative effects in a three-dimensional *in vitro* model of CRC spheroids. (**A**) Spheroids were cultivated in standard conditions (10% FBS) and treated for 24, 48 or 72 h with either the Veh., 2LC1-6, 2LC1-7, or 2LC1-8. The cytotoxicity of the treatments was evaluated thanks to the CellTox green fluorescent dye. The cytotoxicity percentages results reflect the mean NGFI ± S.D. from CellTox green fluorescent dye, for each condition (n = 3). NGFI was calculated as described in section 2.2.3. (**B**) Effect of 2LC1-6, 2LC1-7, and 2LC1-8 on HCT-116-derived spheroid growth in 10% FBS. The spheroid volume was calculated for each treatment condition and at either 24-, 48- or 72-h, and expressed as a fold change of each endpoint measure, normalized to the initial spheroid volume at Day 0. Results are presented as the mean percentages ± S.D. of the Veh.-treated spheroids for each time-point. Each condition was performed in n = 3 technical replicates (black dots). The dotted black lines are drawn to highlight the effect of the tested formulations compared with the Veh.

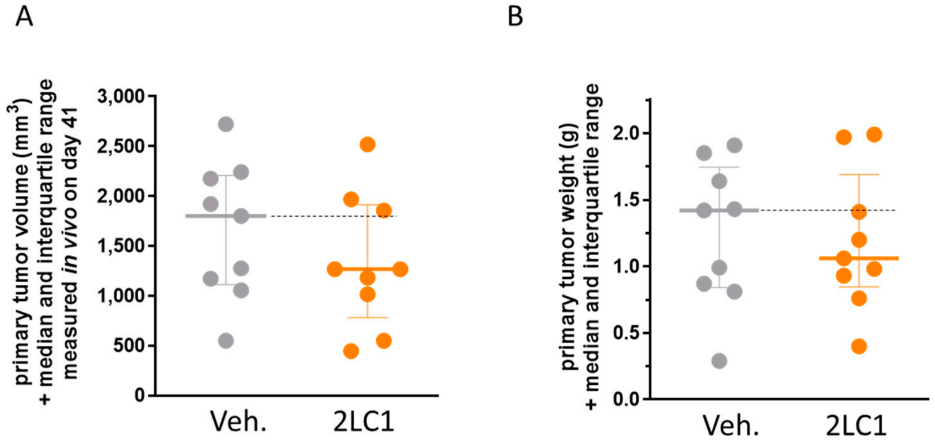

Supplementary Figure S2: The complete sequence of the MIM 2LC1 was tested in an *in vivo* model of CRC cells subcutaneously xenografted. After animals had been randomized on day 15 (D15), the Veh. (grey dots) or the MIM 2LC1 (orange dots) were administered at 100 µL/mouse once daily, *p.o.*, up until day 41 (D41). HCT-116 primary tumor volumes were measured *in vivo* on D41, while primary tumor weights (g), were measured during necropsy on D41. (**A**) The sequential administration of 2LC1 slightly reduces HCT-116 primary tumor growth at D41. Data are represented as individual data points (each point corresponding to a mouse), together with their corresponding median values and interquartile ranges. (**B**) Primary tumor weights (g), measured at necropsy, on D41. Data are given as individual data points together with their corresponding median values and interquartile ranges. The dotted black lines are drawn to highlight the effect of 2LC1 compared with the Veh.

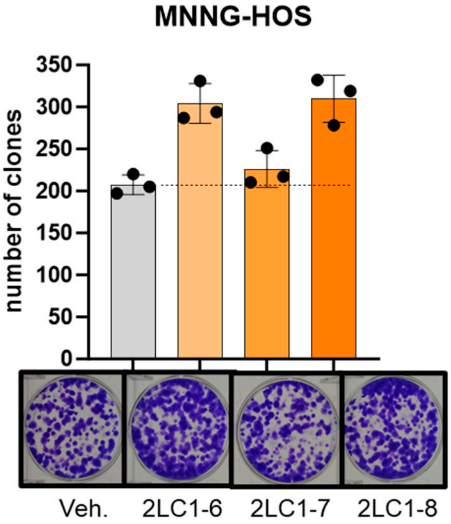

Supplementary Figure S3: Effect of the tested complex MI formulations 2LC1-6, 2LC1-7, and 2LC1-8 on the clonogenic capabilities of MNNG-HOS osteosarcoma cells. The MNNG-HOS osteosarcoma cancer cell line was cultivated during 72 hours in the presence of either the Veh., 2LC1-6, 2LC1-7, or 2LC1-8, before being tested for its clonogenic capabilities. **Upper panel**: the data are presented as the mean ± S.D. of the number of clones counted at the end of the 10-days incubation period, after Crystal Violet staining. The experiment has been done in $n = 3$, each dot representing the cell count obtained in one well of the triplicate. **Lower panel**: representative pictures of the well obtained after clones' staining, taken thanks to a binocular magnifier. The dotted black lines are drawn to highlight the effect of the tested MI formulations, when compared with the Veh.

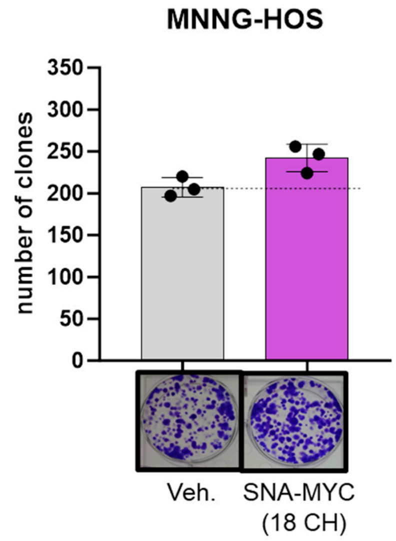

Supplementary Figure S4: Effect of the tested SNA-MYC (18 CH) on the clonogenic capabilities of MNNG-HOS osteosarcoma cells. The MNNG-HOS osteosarcoma cancer cell line was cultivated during 72 hours in presence of either the Veh., or the SNA-MYC (18 CH), before being tested for its clonogenic capabilities. **Upper panel**: the data are presented as the mean ± S.D. of the number of clones counted at the end of the 10-days incubation period, after Crystal Violet staining. The experiment has been done in n = 3, each dot representing the cell count obtained in one well of the triplicate. **Lower panel**: representative pictures of the well obtained after clones' staining, taken thanks to a binocular magnifier. The dotted black lines are drawn to highlight the effect of the tested SNA-MYC (18 CH), when compared with the Veh.

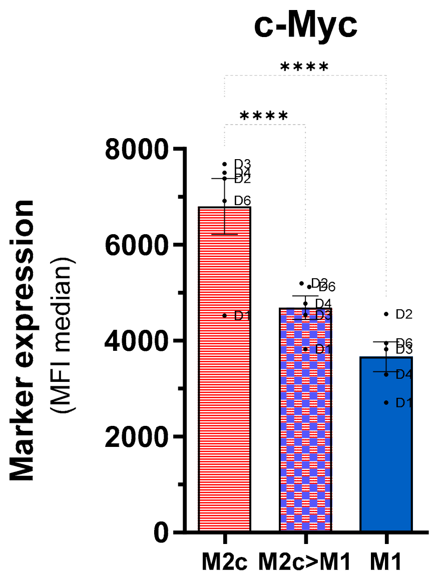

Supplementary Figure S5: C-MYC expression in human CD14⁺-derived M2c, M1 and in "M2c>M1"-switched macrophages. Briefly, PBMCs were retrieved from n = 6 healthy blood donors (D1-D6), separated on Ficoll and cultivated in the presence of M-CSF (50 ng/mL) added with either 20 ng/mL IL-10 (M2c, red histogram), or 20 ng/mL IFN-γ (M1, blue histogram), in order to make them differentiate into the corresponding macrophage subtype. Media were renewed on day 5 and the cytokine used for differentiation were kept the same for M2c and for M1 controls. For the "M2c>M1"-switched macrophage condition (squared red and blue histogram), the 20 ng/mL IL-10 was replaced with 20 ng/mL IFN-γ for the next three days. A boost of LPS (100 ng/mL) was administered on day 7 and the cells were detached, permeabilized and immune-stained for the analysis of C-MYC expression, by flow cytometry, on day 8. The results are presented as the mean ± S.E.M. of MFI values obtained for n = 5 donors (D5 has been excluded from the analysis, due to too few collected cells). Each dot represents the values obtained as the mean of a duplicate measure for each donor. One-way ANOVA, **** $p < 0.0001$ compared with the M2c macrophages.

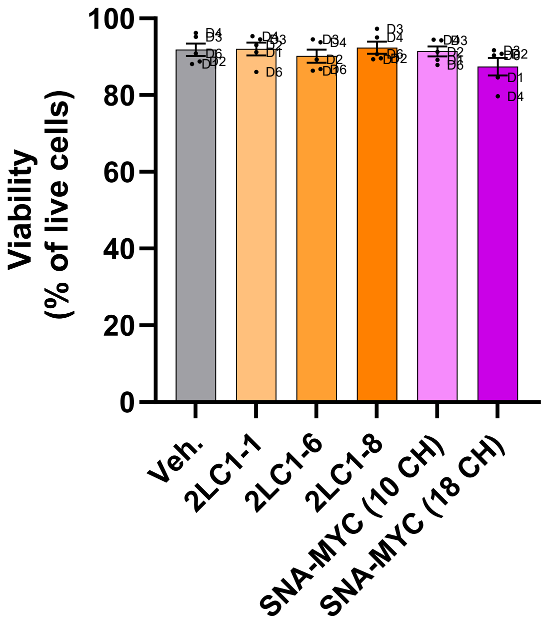

Supplementary Figure S6: The MI formulations from 2LC1, as well as the SNA-MYC employed at 10 CH and at 18 CH do not impact the viability of human CD14^{+}-derived M2c macrophages. Briefly, PBMCs were retrieved from *n* = 6 healthy blood donors (D1-D6), separated on Ficoll and cultivated in the presence of M-CSF (50 ng/mL) and 20 ng/mL IL-10, in order to make them differentiate into M2c macrophage subtype. Media were renewed on day 5 and the tested capsules of 2LC1-1, 2LC1-6, 2LC1-8, SNA-MYC (10 CH), SNA-MYC (18 CH), and the Veh., were incubated with the macrophages for the next three days. A boost of LPS (100 ng/mL) was administered on day 7 and the cells were detached, and stained with Zombie for the analysis of viability, by flow cytometry, on day 8. The results are presented as the mean ± S.E.M. of the percentage of live cells, obtained for *n* = 5 donors (D5 been excluded from the analysis, due to too little collected cells). Each dot represents the values obtained as the mean of a duplicate measure for each donor.

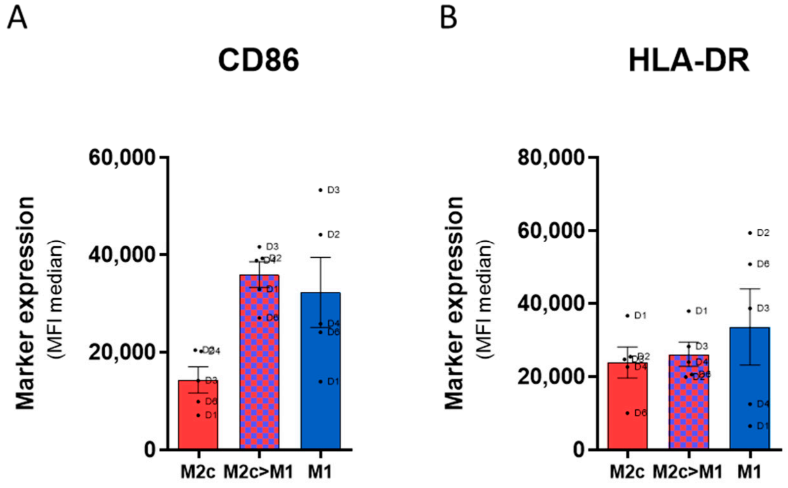

Supplementary Figure S7: CD86 and HLA-DR expression in human CD14⁺-derived M2c, M1 and in "M2c>M1"-switched macrophages. Briefly, PBMCs were retrieved from *n* = 6 healthy blood donors (D1-D6), separated on Ficoll and cultivated in the presence of M-CSF (50 ng/mL) added with either 20 ng/mL IL-10 (M2c, red histogram), or 20 ng/mL IFN-γ (M1, blue histogram), in order to make them differentiate into the corresponding macrophage subtype. Media were renewed on day 5 and the cytokine used for differentiation were kept the same for M2c and for M1 controls. For the "M2c>M1"-switched macrophage condition (squared red and blue histogram), the 20 ng/mL IL-10 were replaced with 20 ng/mL IFN-γ for the next three days. A boost of LPS (100 ng/mL) was administered on day 7 and the cells were detached, and immune-stained for the analysis of (**A**) CD86, and (**B**), HLA-DR expression, by flow cytometry, on day 8. The results are presented as the mean ± S.E.M. of MFI values obtained for *n* = 5 donors (D5 has been excluded from the analysis, due to too little collected cells). Each dot represents the values obtained as the mean of a duplicate measure for each donor.

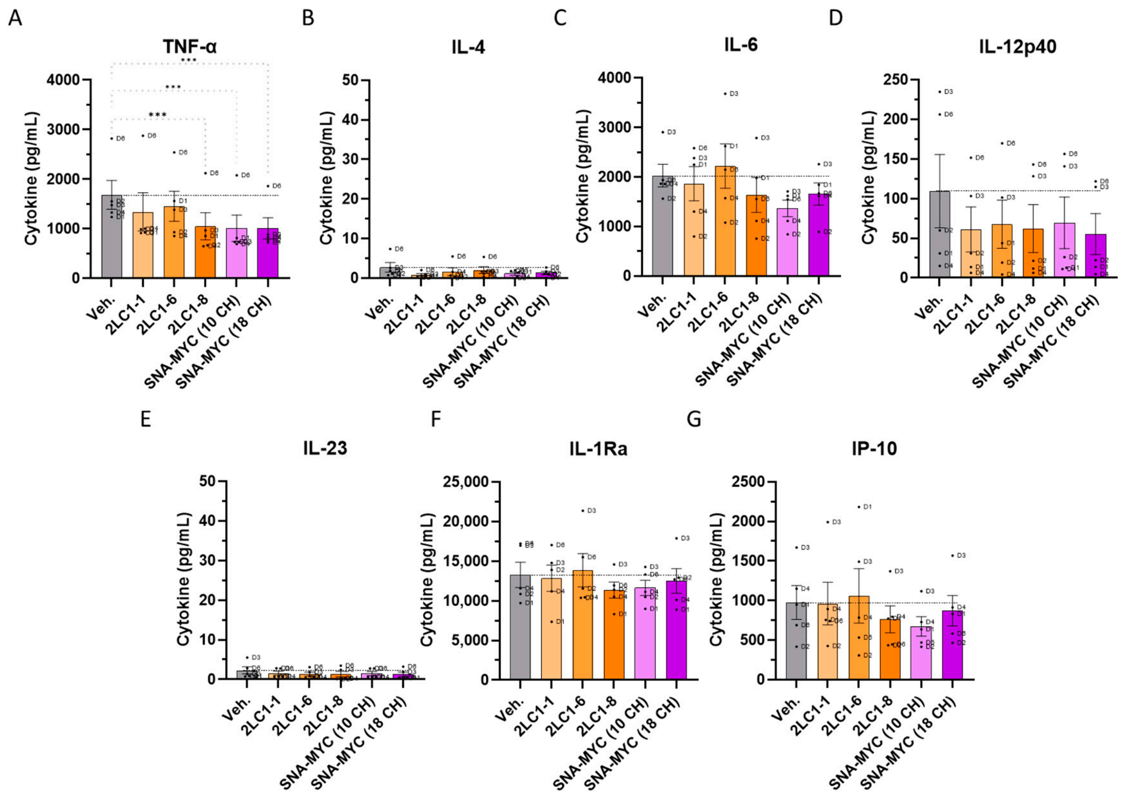

Supplementary Figure S8: The MI formulations from 2LC1, as well as the SNA-MYC employed at 10 CH and at 18 CH reduce the secretion of several macrophages-related cytokines in a model of human $CD14^{+}$-derived M2c macrophages. Briefly, PBMCs were retrieved from n = 6 healthy blood donors (D1-D6), separated on Ficoll and cultivated in the presence of M-CSF(50 ng/mL) and 20 ng/mL IL-10, in order to make them differentiate into M2c macrophage subtype. Media were renewed on day 5 and the tested capsules of 2LC1-1, 2LC1-6, 2LC1-8, SNA-MYC (10 CH), SNA-MYC (18 CH), and the Veh., were incubated with the macrophages for the next three days. A boost of LPS (100 ng/mL) was administered on day 7 and the SNs were collected on day 8 for multiplex assay. (**A-G**) The secretion levels of (TNF-*α*, IL-4, IL-6, IL-12p40, IL-23, IL-1Ra and IP-10) were thus quantified. The results are presented as the mean pg/mL ± S.E.M. of the values obtained for n = 5 donors (D5 has been excluded from the analysis, due to too little collected cells). Each dot represents the values obtained as the mean of a duplicate measure for each donor. The dotted black lines are drawn to highlight the effect of the tested items, when compared with the Veh. One way ANOVA, *** $p < 0.001$, compared with the Veh.-treated M2c macrophages.

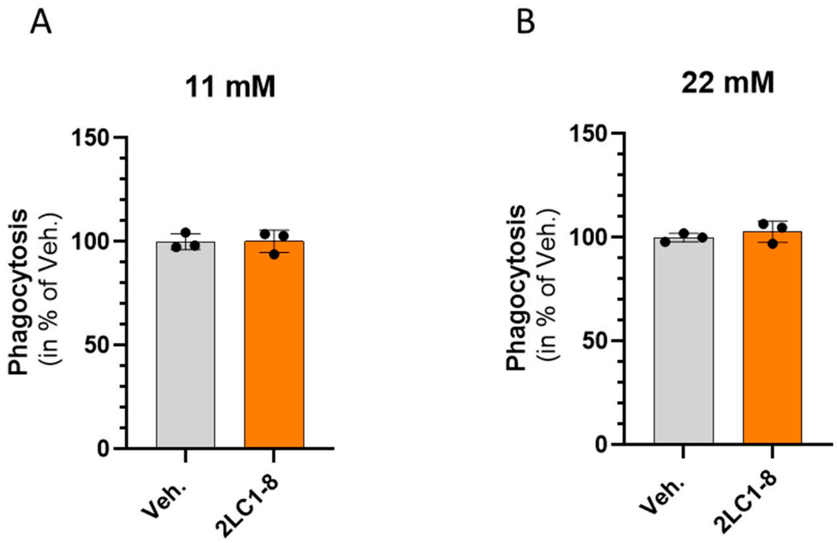

Supplementary Figure S9. 2LC1-8 does not influence the phagocytosis capabilities of human granulocytes. Granulocytes isolated from one healthy donor were treated either with the Veh., or 2LC1-8, either at the final sucrose-lactose concentration of (**A**) 11 mM or (**B**), 22 mM, for 10 min. Fluorescent-beads were then added and incubated with the cells for the next 45 min and the phagocytosed-beads were quantified by flow cytometry. Each condition was performed in $n = 3$ replicates and represented as the mean ± S.D. of the FITC-positive cells, expressed in percentage of the Veh.-treated cells, the latter being set as 100%.
